# In the social amoeba *Dictyostelium discoideum*, density, not farming status, determines predatory success on unpalatable *Escherichia coli*

**DOI:** 10.1186/s12866-014-0328-x

**Published:** 2014-12-20

**Authors:** Susanne DiSalvo, Debra A Brock, jeff smith, David C Queller, Joan E Strassmann

**Affiliations:** Department of Biology, Washington University in St. Louis, St. Louis, Missouri 63130 USA

**Keywords:** Allee effect, Predator–prey, Density dependency, *Dictyostelium discoideum*, *Escherichia coli*

## Abstract

**Background:**

The social amoeba *Dictyostelium discoideum* interacts with bacteria in a variety of ways. It is a predator of bacteria, can be infected or harmed by bacteria, and can form symbiotic associations with bacteria. Some clones of *D. discoideum* function as primitive farmers because they carry bacteria through the normally sterile *D. discoideum* social stage, then release them after dispersal so the bacteria can proliferate and be harvested. Some farmer-associated bacteria produce small molecules that promote host farmer growth but inhibit the growth of non-farmer competitors. To test whether the farmers’ tolerance is specific or extends to other growth inhibitory bacteria, we tested whether farmer and non-farmer amoebae are differentially affected by *E. coli* strains of varying pathogenicity. Because the numbers of each organism may influence the outcome of amoeba-bacteria interactions, we also examined the influence of amoeba and bacteria density on the ability of *D. discoideum* to grow and develop on distinct bacterial strains.

**Results:**

A subset of *E. coli* strains did not support amoeba proliferation on rich medium, independent of whether the amoebae were farmers or non-farmers. However, amoebae could proliferate on these strains if amoebae numbers are high relative to bacteria numbers, but again there was no difference in this ability between farmer and non-farmer clones of *D. discoideum*.

**Conclusions:**

Our results show that farmer and non-farmers did not differ in their abilities to consume novel strains of *E. coli*, suggesting that farmer resistance to their own carried bacteria does not extend to foreign bacteria. We see that increasing the numbers of bacteria or amoebae increases their respective likelihood of competitive victory over the other, thus showing Allee effects. We hypothesize that higher bacteria numbers may result in higher concentrations of a toxic product or in a reduction of resources critical for amoeba survival, producing an environment inhospitable to amoeba predators. Greater amoeba numbers may counter this growth inhibition, possibly through reducing bacterial numbers via increased predation rates, or by producing something that neutralizes a potentially toxic bacterial product.

**Electronic supplementary material:**

The online version of this article (doi:10.1186/s12866-014-0328-x) contains supplementary material, which is available to authorized users.

## Background

Recently, our understanding of the diverse microbial species that constitute a eukaryote's microbiome has been rapidly expanding [1]. Work on this complex network has revealed the importance of microbiome composition, microbial factors, and host responses in mediating the outcome of microbial colonization [2]. Opportunistic pathogens commensally colonize healthy individuals but establish detrimental infections in compromised hosts [3]. In addition, the tolerance or defense towards resident microbes by the intestinal immune system can result in a healthy or inflamed intestinal system [4]. This suggests that the association between specific bacteria and their eukaryotic hosts can result in neutral, beneficial, or pathogenic outcomes that are not always easily predictable or static. Investigating diverse bacteria-eukaryotic interactions has the potential to reveal novel insights into inter-organism relationships. However, teasing apart the effects of eukaryote-bacteria interactions among multicellular hosts with their diversity of bacterial inhabitants can be daunting. Studying these interactions in simple systems, where only a few species interact, may reveal aspects of interspecies interactions difficult to see in studies of more complex microbiota.

The soil dwelling amoeba, *D. discoideum,* is a good model organism to address a variety of biological phenomena because it shares many features with higher eukaryotes and is genetically and biochemically tractable [5]. *D. discoideum* presents an alluring platform to investigate a spectrum of eukaryote-microbe interactions because of its naturally dynamic relationship with bacteria [6-9]. It is a predator of bacteria, a model host for intracellular human pathogens, and a mutualistic partner for different bacterial species [6-9]. Under favorable conditions, *D. discoideum* lives as independent haploid cells that feed on bacteria. When food is sufficiently scarce, amoebae co-aggregate into a motile multicellular slug that seeks out a suitable location for the formation of fruiting bodies [5]. As fruiting bodies form, approximately 20% of the cells die to form a long thin stalk that the rest of the cells ascend. At the tip of the stalk, the remaining cells form a globular structure called the sorus and differentiate into spores. This strategically positions spores for contact and dispersion by passing animals [10]. Once seeded into a new environment, spores hatch into vegetative amoeba and the cycle continues. Additionally and separately, *D. discoideum* can undergo a meiotic sexual cycle to produce genetically diverse haploid progeny [5,11].

In addition to eating bacteria, *D. discoideum* can form symbiotic associations with some bacterial species. This trait appears to be binary, with some amoebae, farmers, consistently carrying bacteria, while others, non-farmers, do not. Farmer clones pick up and carry bacteria through their social and dispersal stages and sporulation and can be identified by the presence of bacteria in their sorus [8]. Carrying edible bacterial species through the social stage enables spores to carry their preferred food source with them to a new environment. Interestingly, farmers also associate with non-edible bacteria. Inedible bacteria can also confer a growth advantage to their hosts by producing compounds that are beneficial to their farmer hosts but toxic to non-farmer competitors [12,13]. Thus farmers have the capacity to cope and flourish with their bacterial passengers and their byproducts even when these are inhibitory to non-farmers of the same species.

The evidence that farmers are resilient to the detrimental effects of their carried bacteria may indicate that farmers are generally less vulnerable to bacterial virulence than their non-farmer counterparts. If true, farmer amoeba should show a higher survival capacity than non-farmers when exposed to different bacterial pathogens and their diverse products. Alternatively, it is possible that farmers have specifically adapted to the unique byproducts of their carried bacteria in a manner that is not generally extendable to other bacterial species. In this case, farmers and non-farmers would respond equivalently to the effects of other, non-carried, bacterial species. Alternatively, because farmers take in and harbor live bacteria, they may make themselves more susceptible to bacterial infections than do non-farmers. If farming is the product of reduced protection from bacterial invasion, then farmers should fare worse than non-farmers when exposed to different bacterial pathogens. Thus, comparing the responses of farmers and non-farmers to variably pathogenic, non-carried, bacterial species can increase our understanding of the farming trait and its associated costs and benefits.

Previous studies have shown that interactions between amoebae and bacteria can be strongly determined by cell density. For instance, *Salmonella typhimurium* inhibits *D. discoideum* proliferation on a rich medium but not on a poor medium, implicating higher bacterial densities in mediating bacterial virulence [14]. Adiba et al. found that amoebae formed plaques on some *E. coli* strains only when plated at high amoebae, or low bacteria, cell numbers [7]. Additionally, *D. discoideum* has been shown to grow on some *Pseudomonas aeruginosa* mutants with attenuated virulence only when seeded on bacterial lawns at high starting amoeba numbers [15]. Thus, varying the numbers of *D. discoideum* amoebae and bacterial cells aids in the determination of differential bacterial virulence [16]. Interestingly, these effects can be caused by social interactions among microbes [17]. We suggest these population dependent outcomes are Allee effects (where increasing group size correlates with increased individual fitness) [18]. Thus, a difference between farmers and non-farmers may not be absolute, but instead could be manifested as shifted Allee effects; farmer clones might fare better than non-farmers at lower amoeba densities and higher bacterial densities.

In order to examine differential responses of amoeba clones to bacteria, we examined growth and spore production of ten farmer and ten non-farmer wild clones on a panel of *E. coli* strains. In addition to comparing farmer and non-farmer growth on *E. coli,* we also tested for an Allee effect in a quantifiable way by examining the effect of *E. coli* and *D. discoideum* numbers on amoeba growth and sporulation efficiency. The *E. coli* panel comprised 33 strains subdivided into three main groups, laboratory, commensal (isolated from the feces of healthy humans) and extraintestinal pathogenic (isolated from blood or urine of patients with symptomatic infections). A few of these strains have fully sequenced genomes [19,20], and the majority have been characterized for their phylogenetic group [21], the presence of certain extracellular antigens, and virulence genes [22,23]. Additionally, their virulence has been correlated across mouse [24], worm [25], and amoeba model systems [7]. From this study, we found that bacterial and amoebae cell densities, but not amoeba farming status, plays a significant role in amoeba growth and sporulation on different *E. coli* strains*.*

## Results

### *Farmer and non-farmer clones do not differ in their overall growth response on* E. coli *strains*

**Figure 1:**
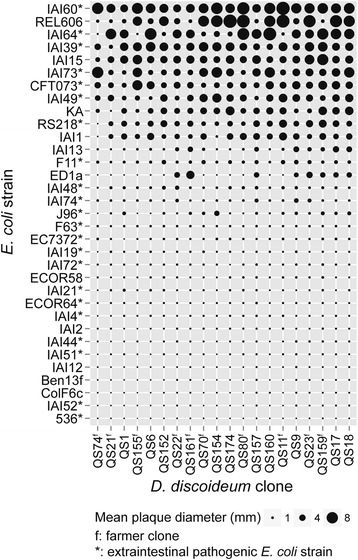
**Both** ***E. coli*** **and** ***D. discoideum*** **identity affect vegetative growth of amoeba** ***.*** Points shows mean plaque diameter four days post-plating for the indicated *E. coli* x *D. discoideum* strain combination. Strains are ordered by mean plaque size. *E. coli* strain identity significantly affects the mean plaque diameters of amoeba clones (χ^2^ = 6930.2, df = 1, P < 0.001).

**Figure 2:**
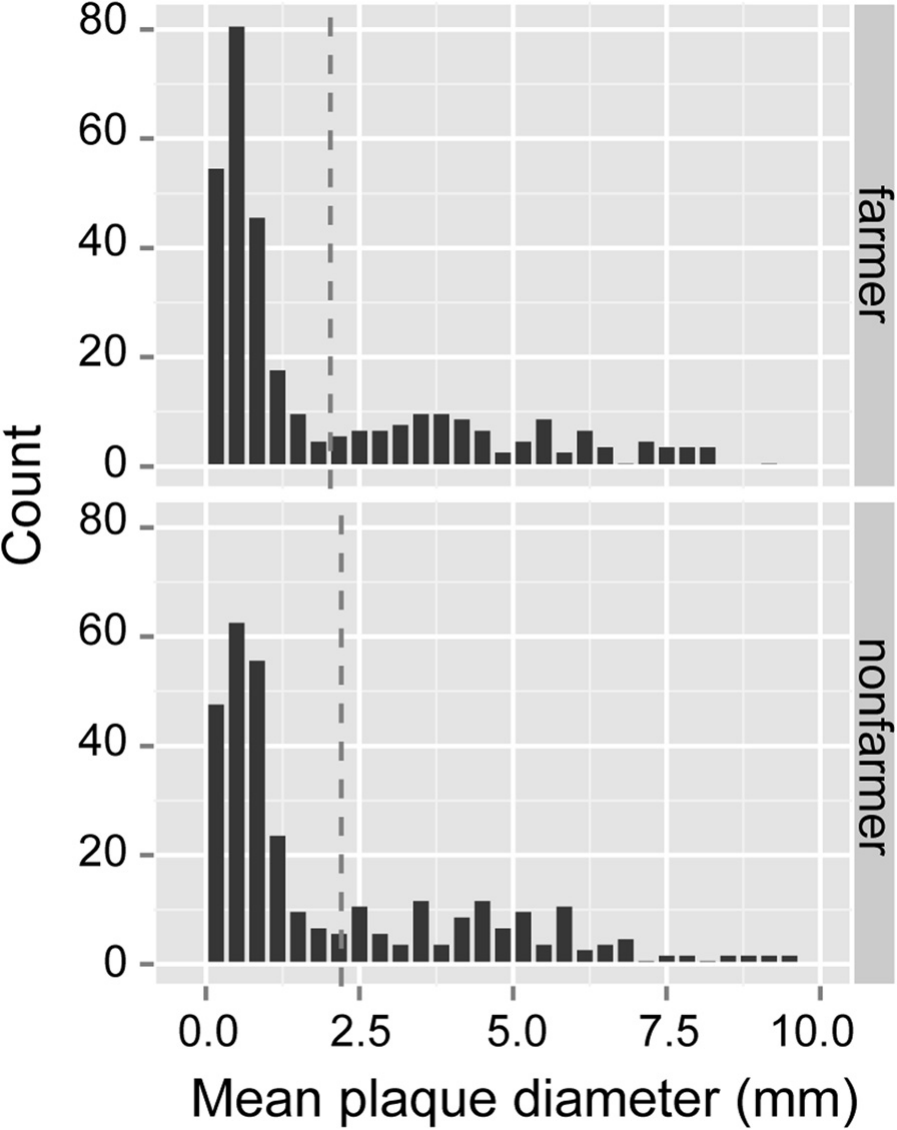
**Farmer and non-farmer amoebae do not differ in their overall plaque diameters on** ***E. coli.*** Figure shows histograms of mean plaque diameter across amoeba clone x bacterial strain combinations. Dashed lines show means of all amoebae x bacteria interactions for farmers (top panel) and nonfarmers (bottom panel). The average mean plaque diameter of farmers on *E. coli* is slightly lower than that of nonfarmers, but these differences are not statistically significant (χ^2^ = 1.02, df = 1, P = 0.31).

**Figure 3:**
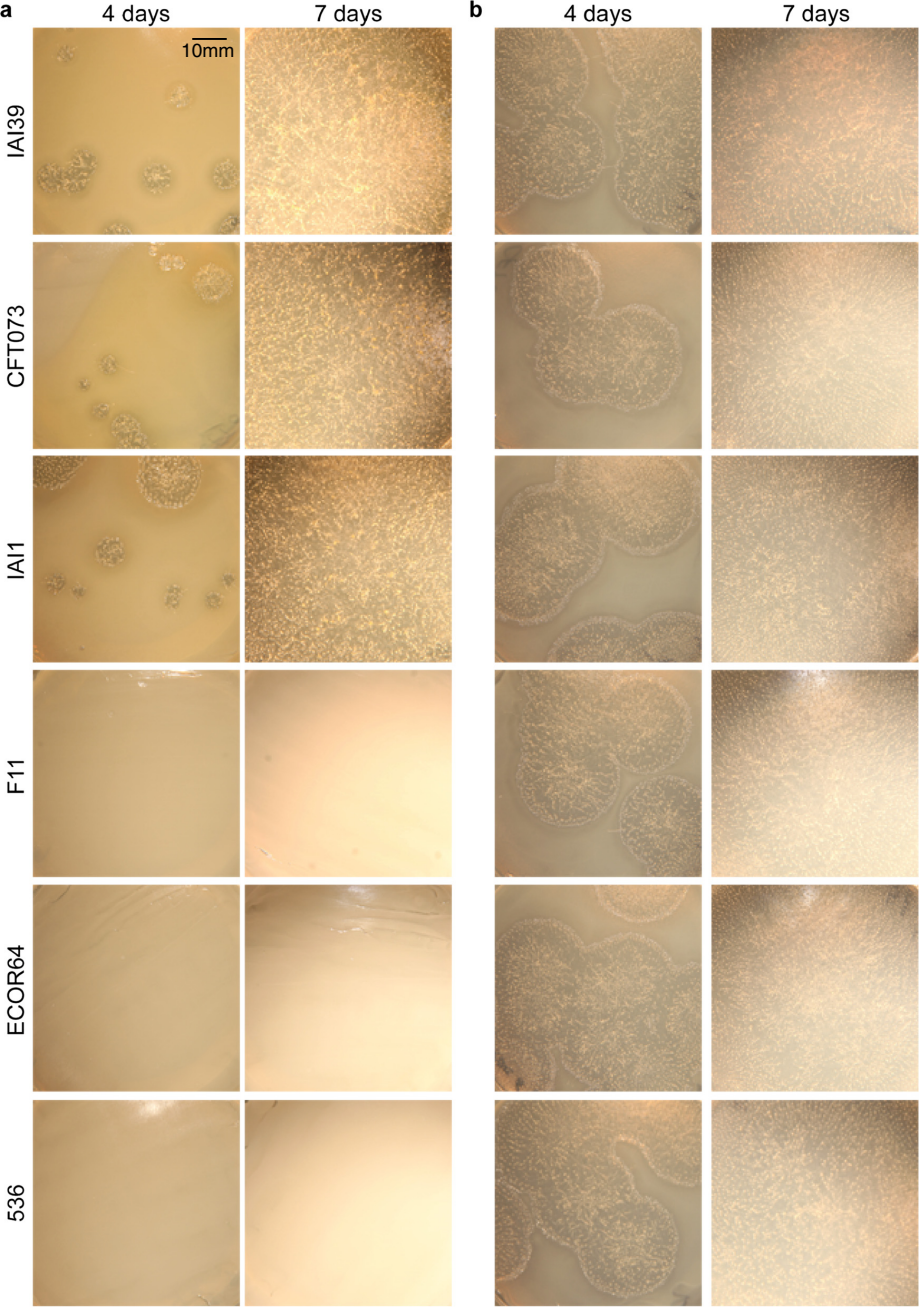
***D. discoideum*** **growth is inhibited on dense lawns of some** ***E. coli*** **strains, achieved by growth on SM medium as compared to SM/5, which has a fifth of some core nutrients (see** **Methods** ***).*** Representative images of spores from *D. discoideum* clone QS9 plated with the indicated bacterial strains on **(a)** SM and **(b)** SM/5, 4 and 7 days post plating. The *E. coli* strains imaged represent a subset of amoeba growth compatible or growth incompatible strains from our collection.

To uncover differences between amoeba clones with respect to their proliferation on *E. coli,* we measured the diameters of lysis plaques (areas cleared of bacteria due to amoeba predation) on bacterial lawns four days after separately plating spores from ten farmer and ten non-farmer clones with each bacterial strain on SM agar. We measured the mean plaque diameter on each plate for every amoeba/bacteria combination (Figure 1) and compared the means of farmers and non-farmers (Figure 2). Plaques were detectable on all plates, indicating that spores germinated and amoebae were able to consume some amount of bacteria from the lawns to produce a clearance zone. Thus, the diameter of plaques gives us a proxy for the extent of bacterial consumption and amoeba proliferation for each amoeba clone/bacterial strain combination. From this analysis we found that *E. coli* strains vary widely in their ability to inhibit the growth of amoeba clones, with *E. coli* strains significantly affecting amoeba clone plaque sizes (χ^2^ = 6930.2, df = 1, P < 0.001). While there was also an effect of amoeba clone on plaque size (χ^2^ = 637.76, df = 1, P < 0.001) the extent of growth and fruiting body formation on each *E. coli* strain appeared to be consistent across clones. For instance, we observed that all amoeba clones grow and produce fruiting bodies on some *E. coli* strains but only form small plaques (generally less than 1 mm) that do not progress to fruiting bodies on a subset of strains (Figure 3a). For these *E. coli* strains plaques remain small and fruiting bodies were never observed even when plates were retained for several weeks, suggesting that they completely prevent *D. discoideum* from reaching numbers sufficient for the social stage of fruiting body formation.

Importantly, plaque sizes were not significantly different between farmer and non-farmer amoeba clones (χ^2^ = 1.02, df = 1, P = 0.31) (Figure 2). Thus, farmer and non-farmer clones are equivalent in their ability to grow on variably pathogenic *E. coli* strains. This means the farming trait is not associated with a generalized resistance to bacterial inhibition even though farmers are resistant to the toxic effects of their own carried bacteria. Interestingly, we found no significant difference in plaque sizes produced by *D. discoideum* on *E. coli* strains of distinct pathogenicity status (pathogenic versus commensal in humans) (χ^2^ = 0.0642, df = 1, P = 0.8), suggesting that under our conditions the ability of a given *E. coli* strain in this collection to inhibit *D. discoideum* growth is not an indicator of its virulence in humans.

### *Growth medium strongly affects interactions between* E. coli *and* D. discoideum

**Figure 4:**
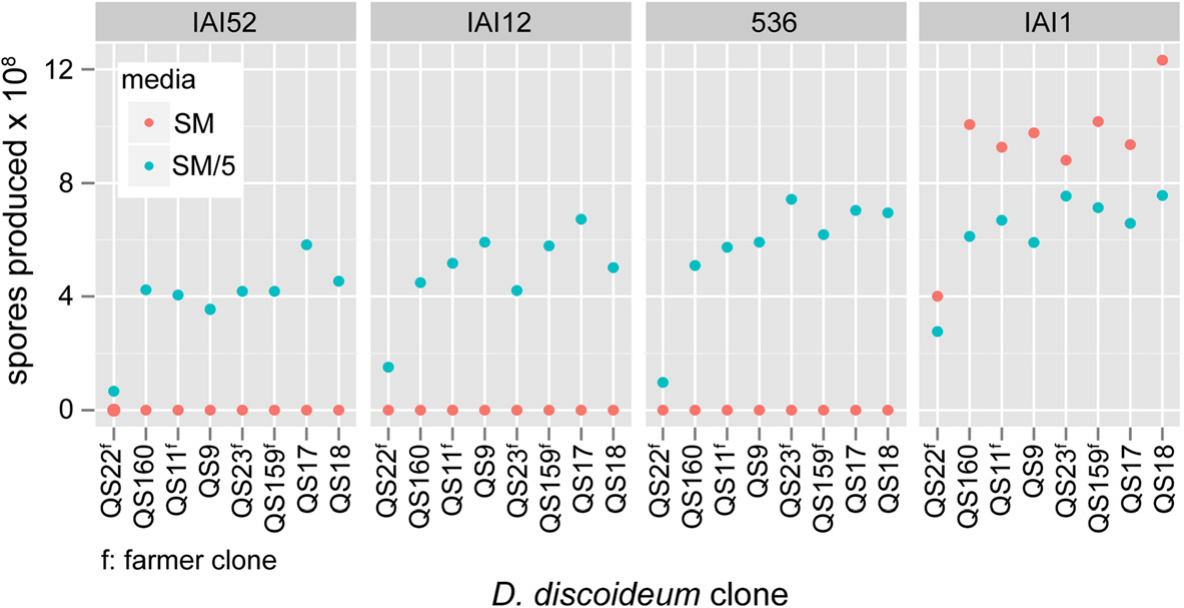
***D. discoideum*** **amoebae produce fewer spores on more concentrated media (SM as opposed to SM/5) with potentially unpalatable bacteria as their food source,** ***E. coli*** **strains IAI52, IAI12, and 536** ***.*** By contrast, they produce more spores on concentrated media with palatable bacteria as their food source, *E. coli* strain IAI1, a significant interaction: χ^2^ = 267.99, df = 4, P < 0.001.

To determine if nutrient richness is important for amoeba growth inhibition by some *E. coli* strains, we examined the growth of amoebae on rich (SM) and poor (SM/5, i.e. 1/5 the glucose, peptone, and yeast extract of SM). We found that all bacterial strains could support amoeba development on SM/5, in contrast to our observation that several strains inhibit amoeba development on SM (Figure 3). To quantify this difference, we compared the spore production of eight amoeba clones when plated with four *E. coli* strains that differentially inhibit amoeba development on our rich (SM) and poor (SM/5) nutrient agar (Figure 4). Both nutrient media and *E. coli* strain significantly affected spore production (media: χ^2^ = 267.99, df = 4, P < 0.001; *E. coli*: χ^2^ = 269.11, df = 6, P < 0.001) with a strong interaction effect (χ^2^ = 203.9, df = 3, P < 0.001).

**Figure 5:**
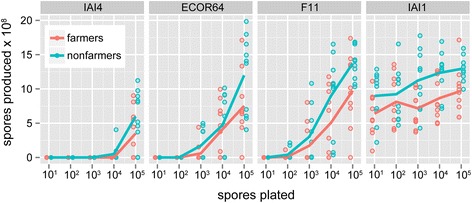
**Amoebae produce more spores when plated at higher densities** ***.*** Points show spores produced by a single *D. discoideum* clone. Lines show mean of ten farmer (red) and ten nonfarmer (blue) clones. Amoebae produce more spores when plated at higher initial spore densities on *E. coli* (χ^2^ = 135.44, df = 4, P < 0.001).

Interestingly, for the more palatable *E. coli* strain IAI1, *D. discoideum* clones were able to produce more spores on rich medium. Thus, for non-inhibitory bacterial food, higher bacterial densities provide more food for amoeba, resulting in greater spore productivity. In contrast, amoebae produced fewer spores on rich medium with the inhibitory *E. coli* strains 536, IAI2, and IAI52 (Figure 4). These results are consistent with those from our plaque assay; the same strains that inhibit plaque formation on rich medium also decrease spore production. As bacterial density increases with increasing nutrient richness, our results suggest that *E. coli* population size can have diverse effects on the number of spores produced by *D. discoideum* during co-culture. Thus, for some *E. coli* strains higher bacterial densities lead to amoeba growth inhibition, while for other strains, higher bacterial densities simply increase the food supply for amoeba predators.

Because bacterial density plays a role in amoeba survival, we wanted to ensure that the ability of specific *E. coli* strains to inhibit amoeba development is not simply explained by ability of these strains to reach higher lawn densities than their non-inhibitory counterparts. To do this, we compared lawn densities and respective *D. discoideum* plaque sizes for a representative subset of strains on SM. We find that although *E. coli* strains produce variable lawn densities (Restricted Likelihood Ratio Test = 10.53, *P* = 7 × 10^−4^) lawn density is not significantly correlated with plaque size (Pearson’s *r* = 0.29, *n* = 12, *P* = 0.36) (Additional file 1). Thus some factor other than final lawn density is responsible for the variation in ameoba development on these bacterial strains.

### *Increasing spore numbers overcomes the toxic effects of* E. coli

Since bacteria numbers appear to play an important role in the effect of *E. coli* on amoeba development, we wanted to determine whether there was also a relationship between amoeba growth on *E. coli* and the starting numbers of amoeba spores. To examine this relationship, we varied initial amoeba spore numbers (from 10^1^ to 10^5^) on SM with four variably inhibitory *E. coli* strains and determined the spore numbers produced by amoebae after development (8 days post-plating). Amoebae overcome the growth inhibition exerted by these *E. coli* strains when amoebae are plated in sufficiently high initial numbers (Figure 5). With increasing initial spore numbers amoebae are more likely to produce spores (χ^2^ = 245.62, df = 4, P < 0.001), and to produce more spores (χ^2^ = 135.44, df = 4, P < 0.001). This experiment further revealed the differential inhibitory effects of *E. coli* strains, as *E. coli* strain identity significantly affected the likelihood of amoeba spore production (χ^2^ = 340.66, df = 4, P < 0.001) and the numbers of spores produced (χ^2^ = 139.94, df = 6, P < 0.001). Spore production was more sensitive to initial plating density for some *E. coli* strains than for others (effect of *E. coli* on slope of spores produced x spores plated: χ^2^ = 71.58, df = 3, P < 0.001). Farmers on average produced fewer spores than non-farmers (χ^2^ = 4.81, df = 1, P = 0.028). Furthermore, non-farmers appear to be slightly more likely to produce spores at lower initial plating densities than farmers, although this effect was not significant (χ^2^ = 135.44, df = 1, P = 0.062).

## Discussion

We found that farmers and non-farmers produce equivalently sized plaques on *E. coli* and are inhibited from forming fruiting bodies on the same *E. coli* strains under the same conditions (Figure 2). These observations suggest that farmers and non-farmers respond similarly to bacterial strains not found associated with farmer clones. Thus, we hypothesize that the enhanced resilience of farmer clones to their own associated bacteria and their secreted compounds stems from a specific adaptation to these agents rather than to a generic resilience to a broad range of bacteria [13,26]. In light of these results, it seems likely that farmers either associate with bacterial strains that they are uniquely compatible with, or that once they associate with a specific bacterial strain, they maintain this association long enough to evolve a tolerance to the potential detrimental effects of the associated strain. Indeed, some bacteria that inhibit the proliferation of some amoeba clones actually enhance proliferation of their carriers, indicating an evolved mutualism [13,26].

Farmers on average produced fewer spores on *E. coli* in our spore density experiment (Figure 5), consistent with previous observations of farmers producing fewer spores when grown on *Klebsiella pneumoniae* [8]. Lower spore numbers are attributed to prudent harvesting by farmers, with farmers switching from the proliferating vegetative stage to the social stage before all of their food has been depleted [8]. It would be interesting to investigate whether this early transition to the social stage is a genetic trait of farmers and the source of their ability to retain residual bacteria as a future food source. Alternatively, this early social transition could be a stress-like response by farmers when colonized by specific bacterial species.

We observed that some *E. coli* strains inhibit amoeba development only when the bacteria are dense, something we achieved by using a rich medium. Increased bacterial densities may lead to bacterial protection from protozoal predation by increasing the concentration of toxic compounds, destroying a resource necessary for amoeba proliferation, and/or by promoting other protective mechanisms. Density dependent processes such as quorum sensing and biofilm formation have been shown to be involved in bacterial virulence and bacterial protection from predation in several systems, including *E. coli* [17,27-33]. Similarly, the aggregation of bacterial or fungal groups may protect individual cells from stressful environments, such as antibiotic exposure, protozoal predation, or immune recognition and phagocytosis [34,35]. Thus, any of these mechanisms may be at play in protecting some *E. coli* strains from amoeba predation when at high densities.

Interestingly, we found that amoebae could overcome the growth inhibition exerted by inhibitory *E. coli* strains on rich medium when amoebae were plated at high starting numbers, demonstrating a positive interaction between amoebae in the solitary stage. Increasing amoeba numbers would increase the total bacterial consumption rate within the environment. Its possible that this effect could reduce bacterial numbers early on such that they fail to reach a critical threshold required for predator defense. Alternatively, amoebae may carry out other protective procedures at high densities or act in mass to produce effective concentrations of compounds that neutralize potential bacterial toxins.

Both bacteria and amoebae proliferate better at higher densities, a positive feedback called the Allee effect. This term comes from Allee et al. (1949), who showed that under certain conditions some species exhibit a positive correlation between population density and individual growth or survival. A strong Allee effect is observed when population growth rate actually becomes negative below a minimal population threshold. Allee effects can arise for several reasons including cooperation among individuals, predator protection, increased mate choice, and protection from weather. Allee effects are typically discussed from an animal, plant, or parasitoid perspectives in behavior, in part due to their importance for conservation and invasive species management [36]. In contrast, this concept has only occasionally been referenced in the general microbial vernacular despite several microbial systems possessing positive density dependent processes, like those regulated by quorum sensing [37]. Since microbes carry out many density dependent processes, it would be interesting to apply them to a similar framework and examine if, and under what conditions, microbial growth could be positively influenced by population size. Social microbes often require specific densities in order to carry out a cooperative trait. For instance it has been reported that some strains of *Myxoccocus xanthus* fail to sporulate when populations fall below a specific threshold [38]. Similarly, the characteristics and efficiency of *Dictyostelium discoideum* aggregation and fruiting body formation is density dependent [39]. Our results add to observations demonstrating the importance of bacterial density in mediating protection from amoeba predation and the ability of large amoeba population sizes in overcoming this protection. Overall, we suggest that the outcome of the interactions between *D. discoideum* with some *E. coli* strains is mediated by a strong Allee effect in the sense that when either species population is below a critical threshold it is less able to protect itself from the detrimental effects of the other organism. Ultimately, this highlights the important role of within-species cooperative interactions in mediating the outcome of interspecies interactions.

Our characterizations of amoeba growth on distinct *E. coli* strains deviate from those of Adiba et al. (2010) for approximately half of the *E. coli* strains. In contrast to the Adiba et al. study, we found no correlation between the ability of amoebas to proliferate on E. coli strains and the pathogenicity of these E. coli strains in humans. Adiba et al. (2010) found that *E. coli* strains IAI13, J96, IAI21, IAI19, IAI4, IAI2, IAI12, and IAI52 supported plaque formation by a lab clone of *D. discoideum* on HL5 medium and so were palatable to amoebae under these conditions [7]. In contrast, we observed that these strains supported only modest plaque formation, but not developmental progression to fruiting bodies, of our wild amoeba clones on SM medium. Additionally, Adiba et al. (2010) reported that strains IAI60, CFT073, IAI73, Rs218, IAI1, and IAI49, inhibited plaque formation by *D. discoideum*, and so were unpalatable to amoebae [7]. In contrast, we observed that these strains supported plaque formation and developmental progression of our wild amoeba clones during co-culture. These incongruities could easily be attributed to differences between amoeba clones, plating strategies, nutrient conditions, and/or laboratory climates used in the two studies. In line with this, we found that plating medium clearly affects the growth of amoeba clones on distinct *E. coli* strains. These differences highlight the importance of specific conditions in the efficacy of using *Dictyoselium discoideum* as a model system to probe bacterial virulence. In any case, these differences do not impact the purpose of our study in addressing the differential growth of amoeba clones on *E. coli* and the influence of each organism’s population size on amoeba growth.

## Conclusions

We found that the inhibitory effect of *E. coli* strains on amoeba plaque size and spore production was dependent on *E. coli* strain identity and on bacterial density. Farming status did not correlate with plaque size on *E. coli*, suggesting that farmer resilience to compounds produced by their symbiotic bacterial species is specific to those bacteria rather than a general response to unpalatable or growth-inhibitory bacteria. Farmers typically produce fewer spores than non-farmers on their food bacteria, presumably because the farming trait favors prudent harvesting and entering the social stage before deep starvation. Starting amoeba population size dramatically affected the ability of amoebae to produce spores when co-cultured with unpalatable bacteria, with increasing amoeba population sizes leading to increased spore production. Our results demonstrate positive Allee effects for both bacteria and amoebae during their antagonistic interactions.

## Methods

### Amoeba clones

All the amoeba clones used for this study were previously described [8]. We ensured the clonality of the samples we used by plating 1 to 10 spores on SM/5 plates with 200 μl of *Escherichia coli* lab strain KA and incubating them for 3 days at 21°C. We then collected amoebae from individual plaques and replated them at 10^5^ amoebae per plate on SM/5 with *E. coli* KA. Once spores were produced on these plates, we collected and froze them in 20% glycerol to create our stocks. Prior to use, we tested each amoeba clone for farming status as previously described [8].

### Bacterial strains

The commensal and extraintestinal *E. coli* strains were previously described [25,40] and were kindly provided to us by Ivan Matic and Sandrine Adiba. The KA *E. coli* strain is used in our laboratory as a common food source for *D. discoideum*.

### Amoeba and bacteria growth conditions

For all the assays, we grew bacterial strains overnight in Luria broth and diluted to a final optical density of 1.5 A_600_ in KK2 buffer. Amoeba spores were mixed with 200 μl of this bacterial culture for the indicated amoeba/strain combination, spread on 30 mL nutrient plates (SM: Formedium™ SM broth SMB0101: 10 g peptone, 1 g yeast extract, 10 g glucose, 1.9 g KH_2_PO_4_, 1.3 g K_2_HpO_4_.3H2O, 0.49 g MgSO_4_, and 17 g agar or SM/5: 2 g peptone, 0.2 g yeast extract, 2 g glucose, 1.9 g KH_2_PO_4_, 1 g K_2_HpO_4_.7H2O, 0.2 g MgSO_4_, 17 g agar), and incubated at 21°C under room lighting.

### Plaque diameter assay

To compare amoeba plaque sizes on each bacterial strain we spread one hundred spores from each amoeba clone with each bacterial sample on SM plates before incubating at 21°C. Four days post plating we measured the diameters of random plaques according to a pre-established grid for each amoeba clone and bacterial strain combination with a graticule under a dissecting scope at 20× magnification.

### Spore counts

We determined total spore numbers eight days post plating amoeba spores with each bacterial strain. For spore counts on SM vs. SM/5, we used 100 spores from 4 farmer and 4 non-farmer clones. For the spore titration experiment, we plated all 10 farmers and 10 non-farmers at the indicated initial spore densities on SM. To harvest spores from each plate we flooded the plate with 5–10 ml KK2 + 0.1% NP-40 and collected the entire surface content of each plate into 15 ml falcon tubes. We then diluted our samples in KK2 and counted spores on a hemocytometer.

### Lawn Density Measurements

We measured lawn densities by growing bacterial lawns on SM for 4 days and resuspending a plug sample from each plate into KK2 for an optical density A_600_ reading. We grew three independent plates for each strain.

### Statistics

We analyzed all data using *R* v3.0.1. We tested the statistical significance of model parameters using likelihood ratio tests on full models fit with and without the parameter of interest. For the plaque diameter assay, we fit linear mixed-effects models to log-transformed spore diameter data using the *lmer* command in the *lme4* package. We modeled *D. discoideum* and *E. coli* strain as mixed effects and modeled farmers vs. non-farmers and pathogenic versus commensal *E. coli* strains as fixed effects.

To test the effect of bacterial density on amoeba growth (SM vs. SM/5 medium), we fit generalized linear mixed models to spore count data using the *glmmadmb* command in the *glmmADMB* package. We used a log link and a negative binomial distribution (to account for overdispersion) with dilution and volume of total spore suspension as offsets. We modeled *D. discoideum* strain as a random effect and medium concentration, *E. coli* strain, and farmers vs. non-farmers as fixed effects.

For the experiment testing the effect of initial spore density, we fit statistical models in two parts: one model for whether or not spores were produced and another model for the number of spores produced. For number of spores produced, we fit linear mixed effects models to spores produced/10^8^ using *lmer*. We modeled *D. discoideum* strain as a random effect and modeled log_10_ (number spores plated), *E. coli* strain, and farmers vs. non-farmers as fixed effects. For whether or not spores were produced, we fit logistic regression models (generalized linear mixed models with binomial distribution) to spore production as binary response using the *glmer* command in *lme4*. Effects were modeled as above.

To test whether there was significant variation among *E. coli* strains in lawn density, we fit a random effects model to log_10_(OD_600_) using the *lmer* command in the *lme4* package. We used log_10_-transformed OD_600_ in order to homogenize variances across strains. We tested the significance of *E. coli* strain as a random effect with a restricted likelihood ratio test (RLRT) using the *exactRLRT* command in the *RLRsim* package. To test whether amoebae created larger plaques on *E. coli* strains with denser lawns, we calculated the Pearson product–moment correlation between mean log_10_(plaque diameter) across *D. discoideum* strains and mean OD_600_ using the *cor.test* command in *R*.

### Ethics statement

As no human or animal subjects were used for this work consent and ethical approval was not required.

## Additional file

Additional file 1: Figure S1. D. discoideum amoebae do not create systematically larger plaques on E. coli that create denser lawns. (a) Variation among E. coli strains in lawn density. Data points show single measurements of turbidity for cells resuspended from plugs of independently grown lawns on SM medium. (b) Variation in plaque size on different E. coli strains. Data points show geometric mean plaque size for each of several D. discoideum clones. (c) No association between lawn density and plaque size. Data show mean ± s.e.m.
