# Supplementary material for: In the social amoeba Dictyostelium discoideum, density, not farming status, determines predatory success on unpalatable Escherichia coli
Source: BMC Microbiol. 2014 Dec 20;14:328. doi: 10.1186/s12866-014-0328-x (PMC4316601; doi:10.1186/s12866-014-0328-x)
Supplement: Additional file 1: Figure S1. — D. discoideum amoebae do not create systematically larger plaques on E. coli that create denser lawns. (a) Variation among E. coli strains in lawn density. Data points show single measurements of turbidity for cells resuspended from plugs of independently grown lawns on SM medium. (b) Variation in plaque size on different E. coli strains. Data points show geometric mean plaque size for each of several D. discoideum clones. (c) No association between lawn density and plaque size. Data show mean ± s.e.m. [file 12866_2014_328_MOESM1_ESM.pdf]

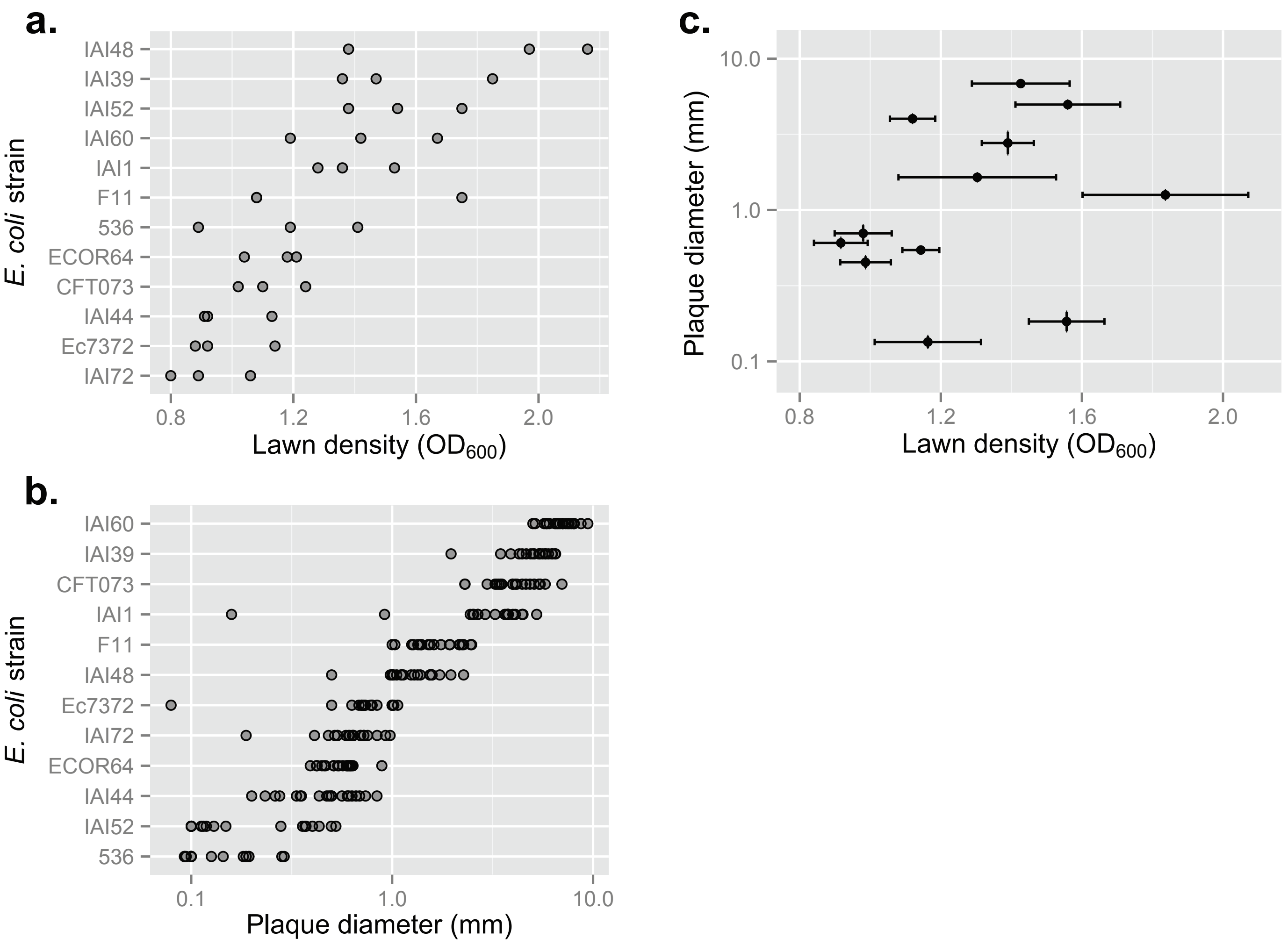

Additional file 1: Figure S1. *D. discoideum* amoebae do not create systematically larger plaques on *E. coli* that create denser lawns. (a) Variation among *E. coli* strains in lawn density. Data points show single measurements of turbidity for cells resuspended from plugs of independently grown lawns on SM medium. (b) Variation in plaque size on different *E. coli* strains. Data points show geometric mean plaque size for each of several *D. discoideum* clones. (c) No association between lawn density and plaque size. Data show mean ± s.e.m.
